# Detection and estimation of *Mastigimas anjosi* (Hemiptera: Calophyidae) populations on *Cedrela fissilis* trees

**DOI:** 10.1098/rsos.211340

**Published:** 2022-03-30

**Authors:** Marcelle C. do N. Prado, Graziella K. F. Giuliani, Thais C. Ghiotto, Janaina B. do Carmo, Júlio C. Guerreiro, Evandro P. Prado, Mario H. F. do A. D. Pogetto, Marcus V. Masson, Wagner de S. Tavares, Carlos F. Wilcken, José C. Zanuncio, Pedro J. Ferreira-Filho

**Affiliations:** ^1^ Programa de Pós-Graduação em Planejamento e Uso de Recursos Renováveis, Universidade Federal de São Carlos, 18052-780 Sorocaba, São Paulo, Brazil; ^2^ Departamento de Agronomia, Centro de Ciências Biológicas, Universidade Estadual de Maringá, 87507-190 Umuarama, Paraná, Brazil; ^3^ Faculdade de Ciências Agrárias e Tecnológicas, Universidade Estadual Paulista ‘Júlio de Mesquita Filho’, 17900-000 Dracena, São Paulo, Brazil; ^4^ Dow AgroSciences, Mogi Mirim Field Station, 13800-970 Mogi-Mirim, São Paulo, Brazil; ^5^ Bracell Ltda., 48030-480 Alagoinhas, Bahia, Brazil; ^6^ Asia Pacific Resources International holdings Ltd. (APRIL), PT. Riau Andalan Pulp and Paper (RAPP), 28300 Pangkalan Kerinci, Riau, Sumatra, Indonesia; ^7^ Departamento de Proteção Vegetal, Universidade Estadual Paulista ‘Júlio de Mesquita Filho’, 18603-970 Botucatu, São Paulo, Brazil; ^8^ Departamento de Entomologia/BIOAGRO, Universidade Federal de Viçosa, 36570-900 Viçosa, Minas Gerais, Brazil

**Keywords:** biological control, forest pest, monitoring, population dynamics, sticky trap, tropical cedar

## Abstract

*Mastigimas anjosi* is an important pest of *Cedrela fissilis* in the southeastern and southern regions of Brazil. The objective of the present study was to investigate the effect of the temperature, relative humidity, rainfall and natural enemies on the flight activity of adults and the movement of *M. anjosi* nymphs, with two sampling methods, yellow sticky traps and direct collections on the leaves of *C. fissilis* trees. The sex ratio of this pest was also assessed. The number of *M. anjosi* individuals was negatively correlated with the minimum and maximum temperatures, with a population peak in late May and early June 2017. The numbers of individuals of this psyllid was positively correlated with the relative humidity and rainfall. Larvae and adults of *Cycloneda sanguinea*, *Hippodamia convergens* adults and *Atopozelus opsimus* nymphs and adults preyed upon *M. anjosi* adults and nymphs on *C. fissilis* leaves in the field. *Mastigimas anjosi* sex ratio was 0.46. Information about sampling methods of *M. anjosi* and its natural enemies is presented and can contribute to the integrated management of this pest in the field.

**Key message**

— The factors favouring infestation of *Mastigimas anjosi* on *Cedrela fissilis* have to be understood for effective control of this pest.
— Important data to promote monitoring and biological control programmes of *Mastigimas anjosi* in *C. fissilis* forests were provided.
— The high numbers of *Atopozelus opsimus*, *Cycloneda sanguinea* and *Hippodamia convergens* individuals in the yellow sticky traps and the predation on *C. fissilis* show the importance of these natural enemies in the integrated management of *M. anjosi*.
— Yellow sticky traps were efficient in sampling of *M. anjosi* adults and its natural enemies and can be used to monitor populations of these insects in the *C. fissilis* plantations.
— Sampling of *Cedrela fissilis* leaves was inefficient to survey insect predators of *M. anjosi*.

## 1. Introduction

The cedar, *Cedrela fissilis* Vell. (Sapindales: Meliaceae), of arboreal size and native to Brazil [1–3], also occurs from Panama and Costa Rica to Argentina [4]. This plant is classified as ‘vulnerable' to extinction in Brazil by the Ministry of the Environment (MMA), possibly due to overexploitation [5,6]. In Brazil, *C. fissilis* occurs in the Amazon, Caatinga, Cerrado and Atlantic Forest biomes, in deep, moist and well-drained soils [7,8]. In addition, this plant is used in the afforestation of parks, public squares and gardens, and in the recovery of degraded areas [9]. The quality of the wood has led to an increase in planting of cedar. However, several insects have been reported as *C. fissilis* pests, including the mahogany shoot borer, *Hypsipyla grandella* Zeller, 1848 (Lepidoptera: Pyralidae) that damage this species, especially in dense plantations without shading [10–12].

The jumping plant-louse, *Mastigimas anjosi* Burckhardt *et al.* [13], (Hemiptera: Psylloidea: Calophyidae), another insect pest of cedar, has been described from individuals collected in Lavras, state of Minas Gerais, Brazil in 2010 on red cedar, *Toona ciliata* M. Roem., 1846 (Sapindales: Meliaceae) trees for the first time [13]. This insect was also reported in Curitiba, state of Paraná, Brazil in 2013 [14], and in Sorocaba, state of São Paulo, Brazil in 2017 on *C. fissilis* trees [15]. *Mastigimas anjosi* is native to Brazil, and the body length of its males and females is 4.8–5.5 mm and 4.2–5.0 mm, respectively, with antennas almost as long as the body and brown. The body colour of *M. anjosi* is light greenish yellow with dark dots and stripes, and its membranous wings with transparent or pigmented pterostigma and veins are used in the identification of this insect [14,16,17].

*Mastigimas anjosi* eggs are yellow and deposited, preferably, on the abaxial surface of young *C. fissilis* leaves. This insect has five instars, and its nymphs suck the raw sap from the veins, leaving the leaves deformed and yellow, which finally dry and drop. These nymphs produce a sugary wax that accumulates in different parts of the leaf, leaflets and petioles, favouring the colonization of sooty mould [14,15]. *Mastigimas anjosi* females lay eggs preferably close to the veins. The nymphs, in numerous populations, cluster in the abaxial surface of the leaves, starting colony formation, close to the midrib. When the physiological conditions of the attacked leaflets are insufficient for the development of nymphs, the psyllids spread throughout the plant, colonizing apical shoots, bark, leaves, petioles and trunk [1,13,17].

The monitoring and sampling can identify factors that influence populations and levels of insect infestation over time, in addition to being important in integrated management [18]. The infested area of *C. fissilis* in the southeastern and southern regions of Brazil by *M. anjosi* has been increasing. The monitoring of this insect and its natural enemies can be performed with yellow sticky traps. In a study conducted in Sorocaba in 2015, a total of 382 *M. anjosi* was collected in the yellow sticky traps, consisting of 249 males and 133 females, from August to November, with the highest numbers captured in August [17]. Other important insect pests of forest plantations are also efficiently sampled and monitored with yellow sticky traps, including the red gum lerp psyllid, *Glycaspis brimblecombei* Moore, 1964 [19–21] and *Euphalerus clitoriae* Burckhardt & Guajará, 2000 (Hemiptera: Psyllidae) [22].

The objectives of this study were to investigate the effect of temperature, relative humidity, rainfall and the presence of natural enemies on the flight activity of adults collected with yellow sticky traps and on the movement of *M. anjosi* nymphs by direct collection on *C. fissilis* leaves. The sex ratio of this insect was also assessed.

## 2. Material and methods

### 2.1. Study site

The population survey of insects was carried out in fragments of seasonal semideciduous forest-type vegetation [23] in an experimental area of the Federal University of São Carlos (UFSCar), Sorocaba campus in Sorocaba (23°30′ S × 47°27′ W, 580 m above sea level) in a transition area between the Cerrado and Atlantic Forest biomes. The insects were collected from August 2016 to July 2017, when the accumulated rainfall was 1544.8 mm. The region climate is humid-subtropical, with rainy summers and dry winters in the transition between the Cwb and Cfa types, according to the Köppen–Geiger climate classification [24]. The average annual temperature was 21.4°C with a maximum of 30.1°C and a minimum of 12.2°C. The predominant soil is of red latosol type [25].

### 2.2. Sampling of *Mastigimas anjosi* adults using yellow sticky traps

*Cedrela fissilis* adult trees were identified based on the analysis of the external morphology of their leaves, fruits and seeds [26] with 16, randomly selected and georeferenced using a Global Positioning System eTrex 10 Garmin^®^ (Lenexa, KS, USA). The diameter of the stem at breast height and the height from the base to the top of the trees ranged from 15.5 to 20.2 cm and from 4.6 to 6.5 m, measured with a tape measure and graduated stick, respectively.

The population survey of *M. anjosi* adults and the natural enemies of this pest was carried out with traps consisting of yellow plastic cards (12 cm long × 10 cm wide) BioControle^®^ (Indaiatuba, state of São Paulo, Brazil) with sticky on both sides and useful area of capture of 100 cm^2^ each, discounting the area for identification of the card [19,20,27,28]. A trap was installed in an upright position, about 20 cm below a lateral branch in horizontal position of the *C. fissilis* tree, tied with a plasticized wire at approximately 2.50 m high to prevent damage by animals and wind, and adjusted with string. Twenty-four collections were performed, with the traps left in the field for approximately 15 days and replaced with new ones at the end of this period. The traps, in each collection, were wrapped with transparent plastic film, packed in brown paper bags identified with the tree number, the collection number and the collection date to avoid damage during transport and storage and to facilitate the visualization of insects in the laboratory.

The traps were sent to the Entomology Laboratory of UFSCar in Sorocaba and maintained at 0.0°C until the identification and counting of *M. anjosi* males and females and the natural enemies of this pest, on the two sides of the traps, under a binocular stereomicroscope M125 C Leica^®^ (Wetzlar, Germany), with lenses with 10× magnification. *Mastigimas anjosi* was identified based on the analysis of its body external morphology [13,16]. The predators were identified according to analysis of their external body morphology and comparison with keys and taxonomic descriptions for the assassin bug, *Atopozelus opsimus* Elkins, 1954 (Hemiptera: Reduviidae) [29], the spotless ladybird beetle, *Cycloneda sanguinea* L., 1763 [30] and the convergent lady beetle, *Hippodamia convergens* Guérin-Méneville, 1842 (Coleoptera: Coccinellidae) [31]. Chrysopidae eggs (Neuroptera) were identified by comparison with their photographs in Brazil [32].

The maximum and minimum temperature (°C), rainfall (mm) and relative humidity (%) values were obtained from the weather station of the Education and Research Meteorological Database (BDMEP), no. 83851 in Sorocaba (23°30′ S × 47°27′ W, 645 m above sea level). This station belongs to the National Institute of Meteorology of the Ministry of Agriculture, Livestock and Food Supply of Brazil. The readings were taken daily, and the average of the maximum and minimum temperatures, accumulated rainfall and relative humidity calculated with the values obtained for the collection dates of the traps.

### 2.3. *Mastigimas anjosi* nymph sampling with direct leaf collections

Two leaves were collected at random from the middle third of each of the 16 *C. fissilis* trees with pruning shears, using a 2.50 m aluminium ladder, per sampling. This pruning shear was attached to the tip of a 4.0 m long aluminium rod, allowing to collect the leaves at high heights. The pruning shears were sanitized with immersion in 2.0% sodium hypochlorite solution in distilled water after cutting each leaf. The leaves collected were individualized in 500 g brown paper bags immediately after the yellow sticky traps had been retrieved and replaced by new ones. These leaves were taken to the Entomology Laboratory at UFSCar in Sorocaba and placed in a biochemical oxygen demand incubator at 14 ± 2°C to reduce their dryness and the movement of the nymphs and to facilitate the identification and counting the insects. The numbers of *M. anjosi* nymphs and natural enemies of this insect, on the abaxial and adaxial surfaces of the leaflets, petiole and midrib of *C. fissilis*, were counted.

### 2.4. Population fluctuation of *Mastigimas anjosi* adults and nymphs

The numbers of adults, captured in the yellow sticky traps, and of nymphs, on *C. fissilis* leaves, of *M. anjosi* were subjected to simple linear regression analysis [33] with the data of the meteorological variables and natural enemies of this pest. The determination of the sampled natural enemies of *M. anjosi* was confirmed with about 30 visits to the field during the day and visual observation of predation (electronic supplementary material).

The data were subjected to analysis of variance (ANOVA) with the entry of variables in the regression model using the RStudio^®^ program at 5% probability [34] (supplier: UFSCar). The data did not show homogeneity of variance and normal distribution and, therefore, were standardized to base 10 logarithm to reduce or equalize the range of values and assist in their interpretation. A correlation matrix between the variables was generated. The Shapiro–Wilk normality test [35] was applied to assess whether the data followed a normal distribution and to verify whether the means differed between treatments and the factors could influence the response variable, directing parametric and/or non-parametric tests. The homogeneity of variances was assessed using the Levene test [36].

The relationship between variables and the visualization of trends in the set, that is, reducing or eliminating the variables number, was verified by principal component analysis (PCA). This analysis was carried out independently of the data distribution and changing only the correlation coefficient, using the Pearson coefficient for data with [37] or without [38] normal distribution. The eigenvalues associated with a component or factor in decreasing order versus the number of components or factor were displayed with the screen plot of their set [39] with the broken-stick model [40].

The regression analysis was performed with the scores resulted from the axes PC1, PC2 and PC3 for adults and PC1 and PC2 for *M. anjosi* nymphs with the model: *Yi* = *β*0 + *β*1*xi* + *€i*, for *i* = 1,…, *n*.

The significant axes for regression analysis PC1 (*p* = 1.027 × 10^−6^), PC2 (*p* = 0.009911) and PC3 (*p* = 4.842 × 10^−6^) for adults and PC1 (*p* = 4.4986 × 10^−6^) and PC2 (*p* = 0.0232) for *M. anjosi* nymphs were considered in the statistics of the model adjustment to identify the explanatory variables with the greatest contribution or predictive power. This was used to determine the linear relationship between the response and the explanatory variables.

### 2.5. Sex ratio of *Mastigimas anjosi*

*Mastigimas anjosi* sex ratio was calculated with the formula: sex ratio = (total of females/total of insects) × 100. The sex of insects was identified by the female's ovipositor [13,15] under a binocular stereomicroscope, with 10× magnification lenses.

## 3. Results

**Figure 1.**
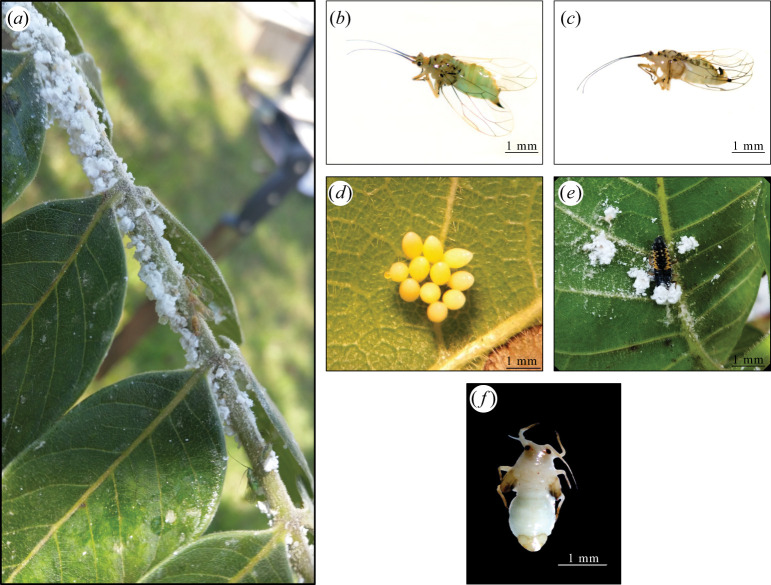
Combined infestation of nymphs and adults of *Mastigimas anjosi* (Hemiptera: Calophyidae) on *Cedrela fissilis* (Meliaceae) (*a*) shoots, (*b*) female, and (*c*) male adults, (*d*) egg cluster, (*e*) predation by *Cycloneda sanguinea* (Coleoptera: Coccinellidae) larva and (*f*) nymph. Sorocaba, state of São Paulo, Brazil. August 2016 to July 2017.

Yellow sticky traps and direct collections on the leaves of *C. fissilis* trees, as sampling methods for *M. anjosi* and its natural enemies, can contribute to the integrated management of this pest in the field. It makes possible to know the flight behaviour of the adults and the movement of nymphs of this insect with the variation of temperature, relative humidity, rainfall and presence of natural enemies. Larvae and adults of *C. sanguinea*, *H. convergens* adults and *A. opsimus* nymphs and adults preyed upon *M. anjosi* adults and nymphs on *C. fissilis* leave in the field (figure 1*a–f*).

**Figure 2.**
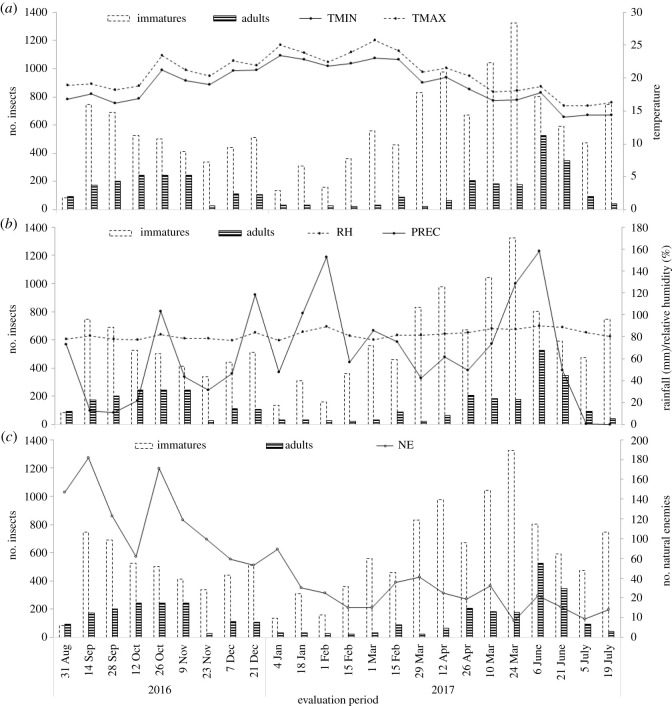
Total number of *Mastigimas anjosi* (Hemiptera: Calophyidae) adults and nymphs per yellow sticky trap and collected, directly, on *Cedrela fissilis* (Meliaceae) leaves, respectively, and (*a*) minimum (TMIN) and maximum (TMAX) temperatures (°C), (*b*) accumulated rainfall (PREC) (mm), relative humidity (RH) (%) and (*c*) natural enemies (NE). Sorocaba, state of São Paulo, Brazil. August 2016 to July 2017.

The total number of adults, captured in the yellow sticky traps, and that of nymphs, on *C. fissilis* leaves, of *M. anjosi* was 3353 and 13 696, respectively (figure 2*a,b*). The number of *M. anjosi* adults and nymphs was 1438 and 4253 individuals, respectively, in late winter and early spring, when the rains were less intense (375 mm) and the minimum and maximum temperatures around 18.8 and 20.5°C, respectively. This number decreased in the summer (247 adults and 1987 nymphs) and increased from the 15th evaluation (15 March 2017) at the beginning of autumn, when the rains were more constant, with cumulative rainfall of 519.8 and 563.6 mm and the minimum and maximum temperatures around 22.7°C and 24.1°C and 17.5°C and 19.1°C, with 1528 adults and 6238 nymphs, respectively (figure 2*a,b*).

The peak in the number of *M. anjosi* nymphs was 1325 insects (41.4 individuals per leaf) in the 20th evaluation (24 May 2017) and that of adults of 527 insects (32.9 individuals per yellow sticky trap) in the next evaluation (7 June 2017), at the end of autumn, with accumulated rainfall of 286.8 mm, relative humidity above 80% and minimum and maximum temperatures of 17.2°C and 18.4°C, respectively (figure 2*a*,*b*).

A total of 1892 individuals of natural enemies was captured with the yellow sticky traps, being 1709 coccinellid (Coleoptera) and 183 reduviid (Hemiptera: Heteroptera) adults, with a peak of 191 insects (11.9 individuals per trap) in the fifth evaluation (26 October 2016) (figure 2*c*).

**Figure 3.**
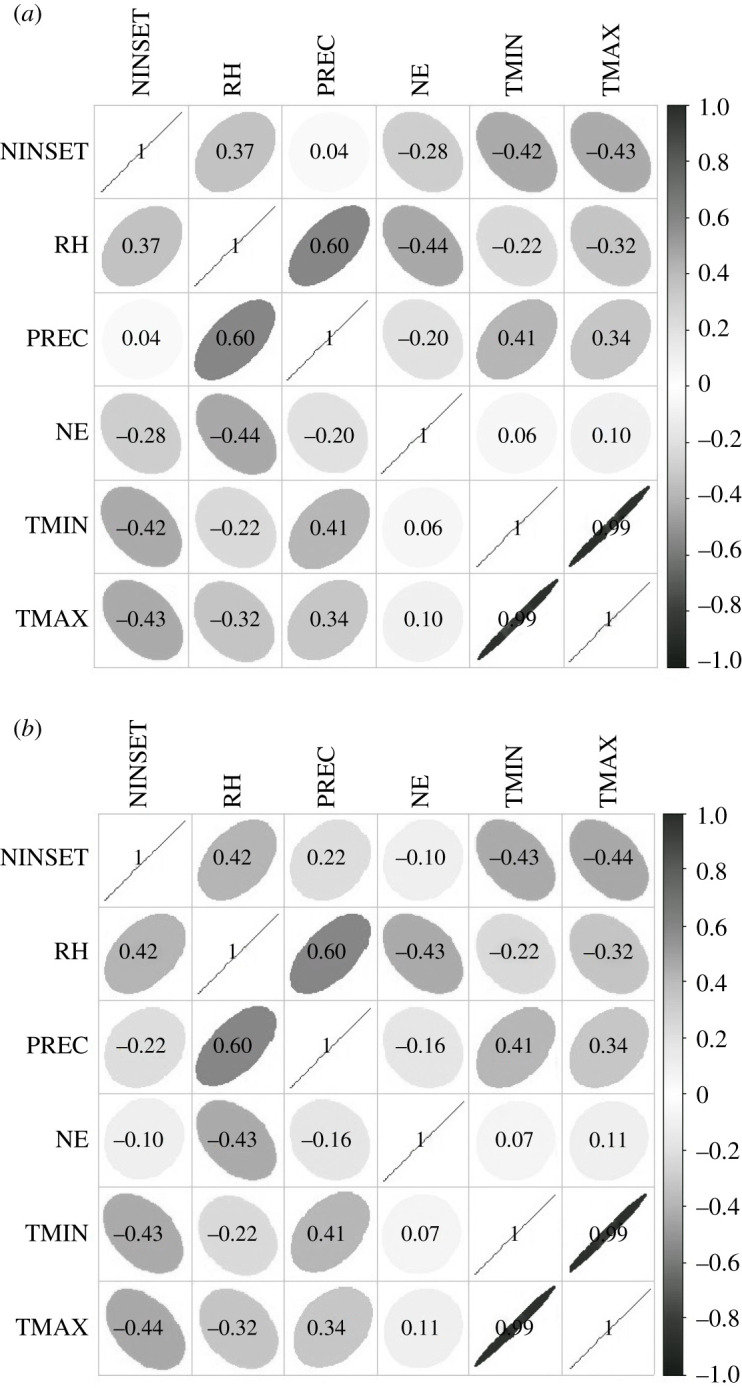
Linear correlation matrix between the response variables insect number in the nymph (NINSET) (*a*) and adult stage (NINSET) (*b*) and the explanatory variables minimum (TMIN) and maximum (TMAX) temperatures (°C), relative humidity (RH) (%), accumulated rainfall (PREC) (mm) and natural enemies (NE). Sorocaba, state of São Paulo, Brazil. August 2016 to July 2017.

The correlation matrix, obtained from the transformed data, showed a linear relationship between the explanatory variables with the populational peak of *M. anjosi* nymphs and adults, in late May and early June 2017. The response variable of nymphs number (NINSET) (*r* = −0.42 and *r* = −0.43) and adults (NINSET) (*r* = −0.43 and *r* = −0.44) of *M. anjosi* correlated, inversely, with the explanatory variables minimum (TMIN) and maximum (TMAX) temperatures, respectively (figure 3*a*,*b*). The number of nymphs (NINSET) (*r* = 0.37 and *r* = 0.04) and adults (NINSET) (*r* = 0.42 and *r* = 0.22) of this psyllid was positively correlated with the relative humidity and rainfall, respectively (figure 3*a*,*b*).

The number of natural enemies was negatively correlated (*r* = −0.28) with that of nymphs (*r* = 0.10) and with those of *M. anjosi* adults (figure 3*a*,*b*). *Atopozelus opsimus* nymphs and adults, *C. sanguinea* larvae and adults and *H. convergens* adults preyed upon *M. anjosi* adults and nymphs on *C. fissilis* leaves in Sorocaba. Chrysopidae eggs were also observed on leaves of this plant.

**Figure 4.**
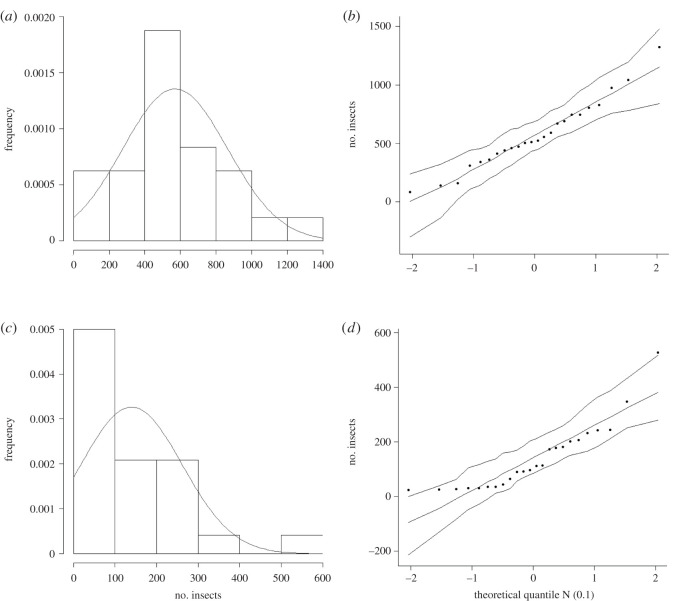
Histograms and QQNormal graphs for the nymph (*a*,*b*) and adult numbers (*c*,*d*) of *Mastigimas anjosi* (Hemiptera: Calophyidae) on *Cedrela fissilis* (Meliaceae) trees. Sorocaba, state of São Paulo, Brazil. August 2016 to July 2017.

The normality test showed that the H_0_ was accepted for the *M. anjosi* nymphs population, *W*_calculated_ = 0.9692 > *W*_(0.05;24)_ = 0.905 with the *p*-value calculated by *p*[*W* > *W*_calculated_] = 0.6478 > *α* = 0.05. This shows that it is possible to affirm, with a 5% significance level, that the distribution of the data in this sample is normal. However, this was rejected for *M. anjosi* adults, *W*_calculated_ = 0.8404 < *W*_(0.05;24)_ = 0.905, with *p*-value calculated by *p*[*W* < *W*_calculated_] = 0.0014 < *α* = 0.05 (figure 4*a*,*b*). The hypothesis that data on population variances are similar (homogeneity) was rejected, that is, the means and medians of the *M. anjosi* nymph and adult populations differ according to ANOVA (*p* = 1.362 × 10^−36^) and the Kruskal–Wallis test (*p* = 2.2 × 10^−16^), respectively.

**Figure 5.**
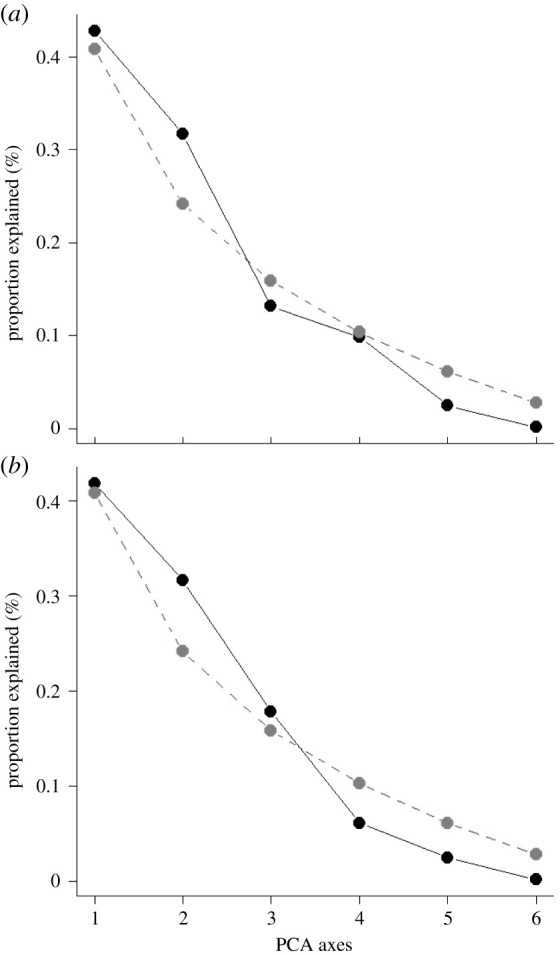
Broken-stick model for the response variables nymph (*a*) and adult (*b*) numbers of *Mastigimas anjosi* (Hemiptera: Calophyidae) and explanatory variables minimum and maximum temperatures (°C), relative humidity (%), accumulated rainfall (mm) and natural enemies of principal component analysis (PCA). Sorocaba, state of São Paulo, Brazil. August 2016 to July 2017.

**Figure 6.**
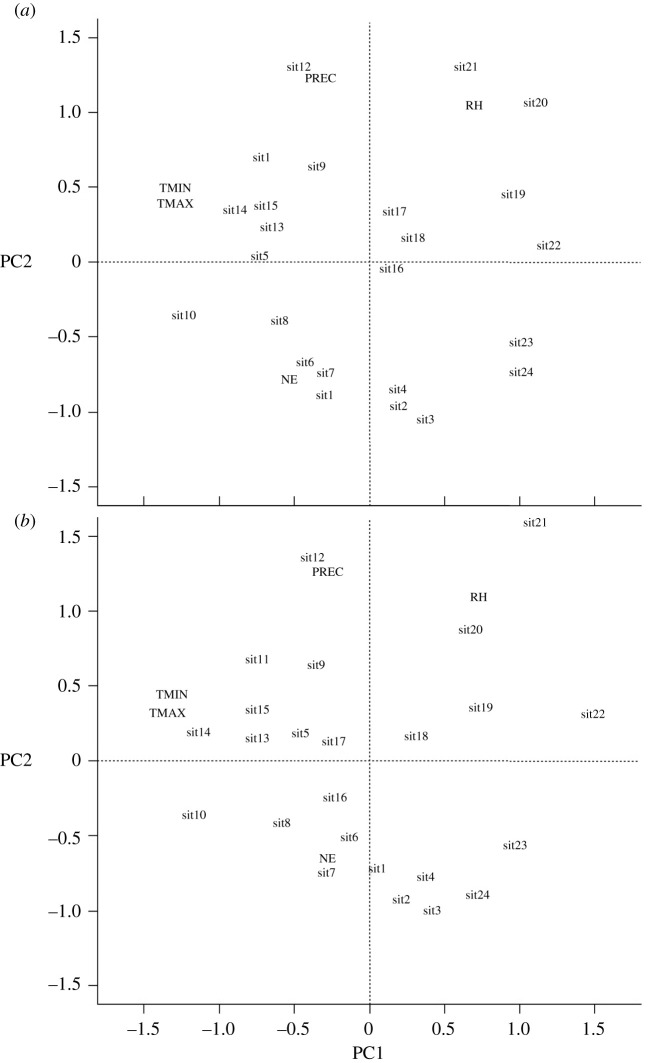
Principal component of main component analysis (PCA) containing the explanatory variables minimum (TMIN) and maximum temperatures (TMAX) (°C), relative humidity (RH) (%), accumulated rainfall (PREC) (mm) and natural enemies (NE) for *Mastigimas anjosi* (Hemiptera: Calophyidae) (*a*) nymphs and (*b*) adults. Sorocaba, state of São Paulo, Brazil. August 2016 to July 2017. sit = evaluations number.

**Figure 7.**
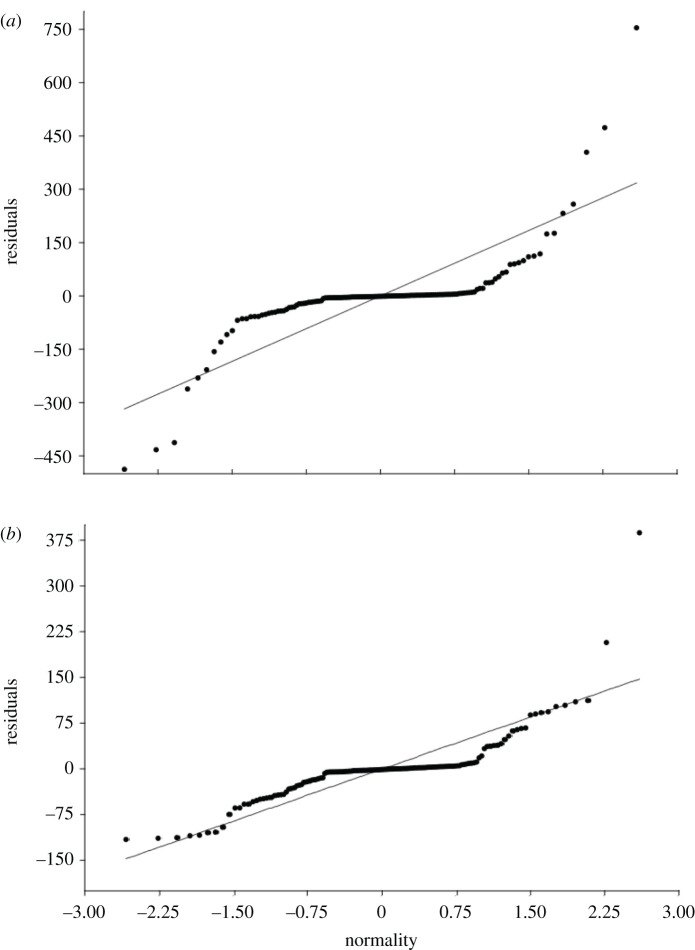
Residual analysis obtained from the scores related to the simple linear regression equations, characterized by the comparison between the values of the response variables number of *Mastigimas anjosi* (Hemiptera: Calophyidae) nymphs (*a*) and adults (*b*) and the explanatory variables minimum and maximum temperatures (°C), relative humidity (%), accumulated rainfall (mm) and natural enemies on *Cedrela fissilis* (Meliaceae) trees. Sorocaba, state of São Paulo, Brazil. August 2016 to July 2017.

The PCA was correlated with the response variable and the PC1 and PC2 axes set for nymphs and PC1, PC2 and PC3 for adults, with a higher eigenvalue of one, 42.79% and 31.68% for nymphs and 41.88%, 31.61% and 17.83% for *M. anjosi* adults, respectively, from the variation of the dataset (figure 5*a*,*b*). The PCA retained all factors with eigenvalues higher than one (table 1), identifying the variables per axis. The explained proportion of variability was low for the PC3 (13.14%), PC4 (9.79%) and PC5 (2.45%) axes for nymphs and the PC4 (6.09%) and PC5 (2.44%) for adults. The PC1 and PC2 axes, for nymphs, and the PC1, PC2 and PC3 for *M. anjosi* adults in the broken-stick graph indicated most of the variability, with a more pronounced decrease from the PC3 axis (12.6%) for nymphs and PC4 (5.2%) for adults of this pest (figure 6*a*,*b* and table 1). The correlation between the variables and the axis of PC1 was high and inversely proportional to the minimum (*r* = −0.91) and maximum (*r* = −0.93) temperatures for nymphs (*r* = −0.93) and adults (*r* = −0.97) of *M. anjosi*, respectively, moderate and direct for relative humidity (*r* = 0.50; *r* = 0.42) and weak and inversely proportional for rainfall (*r* = −0.19; *r* = −0.26) and natural enemies (*r* = −0.36; *r* = −0.26), respectively (figure 7*a,b* and table 2). The PC1 axis showed a greater relationship between the response variable number of *M. anjosi* nymphs and adults (NINSET) and the explanatory minimum (TMIN) and maximum (TMAX) temperatures with the population fluctuation of this psyllid. The number of nymphs (*r* = 0.88 and *r* = 0.75) and adults (*r* = 0.90 and *r* = 0.72) of *M. anjosi* correlated with the variables rainfall and relative humidity and the axis of PC2, high and direct correlation, respectively, in addition to low and inversely proportional correlation with natural enemies (*r* = −0.55; *r* = −0.40) (figure 7*a,b* and table 2).

**Table 1.** Scores resulting from principal component analysis (PCA) containing the eigenvalues, proportion explained and cumulative proportion by main component of the variables for *Mastigimas anjosi* (Hemiptera: Calophyidae) nymphs and adults.

stages	parameters	PC1	PC2	PC3	PC4	PC5
nymphs	eigenvalue	2.5676	1.9010	0.7882	0.5876	0.1473
	proportion explained	0.4279	0.3168	0.1314	0.0979	0.0245
	cumulative proportion	0.4279	0.7447	0.8761	0.9740	1
adults	eigenvalue	2.5130	1.8967	1.0700	0.36576	0.1464
	proportion explained	0.4188	0.3161	0.1783	0.06096	0.0244
	cumulative proportion	0.4188	0.7349	0.9133	0.97422	1

**Table 2.** Correlation between variables with axes of principal component analysis (PCA) for *Mastigimas anjosi* (Hemiptera: Calophyidae) nymphs and adults on *Cedrela fissilis* (Meliaceae) trees. NINSET, insect number in the nymph and adult stages; RH, relative humidity (%); PREC, accumulated rainfall (mm); TMIN, minimum temperature (°C); TMAX, maximum temperature (°C) and NE, natural enemies.

Variable	PC1	PC2	PC3	PC4	PC5	PC6
*Mastigimas anjosi* nymphs						
NINSET	0.68347	0.24471	0.21660	−0.652498	−0.017347	−2.05 × 10^3^
RH	0.50464	0.75411	−0.29247	0.161299	−0.255009	7.89 × 10^3^
PREC	−0.19323	0.87776	−0.35780	−0.059123	0.246317	−5.23 × 10^2^
TMIN	−0.90557	0.35786	0.15486	−0.111814	−0.106688	−6.32 × 10^4^
TMAX	−0.92616	0.27208	0.19045	−0.150248	−0.071724	6.47 × 10^4^
NE	−0.36134	−0.54758	−0.68368	−0.311970	−0.069464	−4.23 × 10^1^
*M. anjosi* adults						
NINSET	0.59478	0.039130	0.598260	−0.34695	−0.036521	−0.016521
RH	0.42810	0.726125	−0.163149	0.25625	−0.351098	−0.124864
PREC	−0.26260	0.902608	0.112173	0.11043	0.130434	−0.079130
TMIN	−0.93933	0.355294	−0.002609	−0.12307	−0.086975	0.212220
TMAX	−0.97238	0.235268	0.018699	0.13133	−0.090454	0.120895
NE	−0.26657	−0.403131	0.790171	0.25962	−0.012176	0.287018

The variable natural enemies, in the PC3, showed a moderate and direct correlation (*r* = 0.79) with the *M. anjosi* adult numbers (table 2). The adjusted coefficient of determination for nymphs (*R*^2^ adjusted = 0.13161; *n* = 24) and adults (*R*^2^ adjusted = 0.28997; *n* = 24) of *M. anjosi* showed the fit of the regression analysis line with the dataset (figure 7*a*,*b*).

The relationship between the variables analysed, the normality of the data and the F value (*F* = 1.6972) for nymphs and adults (*F* = 2.8786) of *M. anjosi* was significant, but reduced (table 3).

**Table 3.** Linear regression model of Adrien-Marie Legendre (1805) from the principal components analysis (PCA) with the PC1 and PC2 axis for the response variables insect numbers (NINSET) in the nymphs stage and PC1, PC2 and PC3 axis for *Mastigimas anjosi* (Hemiptera: Calophyidae) adults on *Cedrela fissilis* (Meliaceae) trees.

response variables	insect numbers	*R*^2^ adjusted	*F*	d.f.1, d.f.2	*p-*value
NINSET nymphs	24	0.13161	1.6972	5.18	0.0420
NINSET adults	24	0.28997	2.8786	5.18	0.0011

*Mastigimas anjosi* sex ratio was 0.46, with 1526 females and 1827 males captured in yellow sticky traps.

## 4. Discussion

The attractiveness of *M. anjosi* and other hemipterans to the yellow sticky traps is due to its yellow colour, with a wavelength of electromagnetic radiation between 500 and 600 nm and peak at 550 nm, similar to that of green leaves such as those of *Acacia cyanophylla* Lindley (Fabales: Fabaceae), sea pumpkin, *Arctotheca nivea* (L. fil.) Lewin (Asterales: Asteraceae), parrot bush, *Banksia sessilis* Knight (Proteales: Proteaceae) and garden pea, *Pisum sativum* L. (Fabales: Fabaceae) in Australia [41], as preferred places for food and oviposition, compared to green and yellow, red, navy blue, black, magenta and true blue colours for the vegetable leafhopper, *Austroasca viridigrisea* Paoli, 1936 (Hemiptera: Cicadellidae) adults in Narrabri, New South Wales, Australia [42]. Green, blue and yellow reflect considerable radiation, in the range of 500–560 nm, but with greater reflectance for yellow to adults of the boronia psyllid, *Ctenarytaina thysanura* Ferris & Klyver, 1932 (Hemiptera: Aphalaridae) in Hobart, Tasmania, Australia [43]. In addition, diurnal insects respond to energy in the visible spectrum range from 350 to 650 nm, as observed for the black bean aphid, *Aphis fabae* Scopoli, 1763, the cabbage aphid, *Brevicoryne brassicae* L., 1758 and the green peach aphid, *Myzus persicae* Sulzer, 1776 (Hemiptera: Aphididae) in a laboratory in England [44]. The greater number of nymphs than adults collected may be due to the direct collections of the first on the leaves in groups as a defensive behaviour against natural enemies [1,45]. The greater numbers of *M. anjosi* nymphs and adults in the autumn confirm the effect of climatic conditions on the capture of hemipterans, as reported for the highest number of individuals of sharpshooters, *Acrogonia citrina* Marucci & Cavichioli, 2002, *Bucephalogonia xanthophis* Berg, 1879 and *Dilobopterus costalimai* Young, 1977 (Hemiptera: Cicadellidae) during periods of high rainfall (207.8 mm) in November 2003 in Viçosa, state of Minas Gerais, Brazil [46].

The peak of *M. anjosi* nymphs population in the 20th evaluation and that of adults in the following is due to the nymphs of this insect completing the development cycle in approximately 20 days [47] and the low number of natural enemies captured during the period, favouring the development of nymphs and, therefore, the highest number of adults. The high number of *M. anjosi* individuals, collected in Sorocaba, in May and June, a period with rainfall above 100 mm after the dry period [48], confirms the beneficial effect of this factor on the development of *C. fissilis* plants and, consequently, in the reproduction and development of this insect [22].

The highest number of individuals of natural enemies, collected in October, follows the same trend as the *M. anjosi* number due to the population dynamics of natural enemies being directly related to that of their prey or hosts. Larger pest populations support a greater number of natural enemies due to the increased supply of food/host, as observed for onion thrips, *Thrips tabaci* Lindeman, 1889 (Thysanoptera: Thripidae) and their predators from five families, including Aeolothripidae (Thysanoptera) on common onion plants, *Allium cepa* L. (Asparagales: Amaryllidaceae), with population peaks, respectively, in late July and early August in the central and western regions of New York, USA in 2011 and 2012 [49].

The inverse linear correlation between the explanatory variables minimum (TMIN) and maximum (TMAX) temperatures with the *M. anjosi* nymphs and adults numbers with a population peak in late May and early June was similar to that reported for *G. brimblecombei* nymphs per *Eucalyptus* L'Hér. (Myrtales: Myrtaceae) leaf in Botucatu and São Simão, state of São Paulo, Brazil [44]. The correlation between the *G. brimblecombei* adult numbers and temperature was also inverse in collections with yellow sticky traps on river redgum, *Eucalyptus camaldulensis* Dehnh, 1832 trees in January to June 2005 in Luiz Antônio, state of São Paulo, Brazil [19] and for the largest number of *M. anjosi* individuals collected on *C. fissilis* trees in August 2015 during a period with low rainfall and high temperatures with yellow sticky traps in Sorocaba [15]. Lower numbers of *M. anjosi* individuals, captured in the summer, and higher in the winter months, characterize the impact of temperature on the population dynamics of this insect.

The numbers of natural enemies, negatively correlated with that of *M. anjosi* nymphs, indicate their greater preference to feed on nymphs than on adults of this pest, because they are smaller, apterous and low mobile [4,16]. The presence of *A. opsimus*, *C. sanguinea* and *H. convergens* preying upon *M. anjosi* on *C. fissilis* leaves in Sorocaba shows the importance of these natural enemies as reported for those preying the Asian citrus psyllid, *Diaphorina citri* Kuwayama, 1980 (Hemiptera: Liviidae) on *Citrus* (Sapindales: Rutaceae) in China [50] and the guava psyllid, *Triozoida limbata* Enderlein, 1918 (Hemiptera: Triozidae) on common guava, *Psidium guajava* L. (Myrtales: Myrtaceae) in Petrolina, state of Pernambuco, Brazil [51]. *Atopozelus opsimus*, Chrysopidae and Syrphidae species are generalist natural enemies, and they were reported preying *M. anjosi* on *T. ciliata* trees in 2013 and 2015 in Conselheiro Lafaiete and Ouro Branco, state of Minas Gerais, Brazil [52]. *Atopozelus opsimus* preyed upon *M. anjosi* nymphs and adults on *C. fissilis* trees in Montes Claros, state of Minas Gerais, Brazil, and their nymphs feed on honeydew produced by this pest. This predator did not prevent damage to *C. fissilis*, which may be related to the high temperatures in this region, reducing its reproduction and increasing its development period [1].

The H_0_ hypothesis acceptance shows normal distribution of data on the *M. anjosi* nymph numbers and that their base can be accepted and compatible with approximately 65% of the residue data and that the W value should be rejected for the adult numbers of this insect. On the other hand, the lack of normality in the frequency distribution of this dataset may be related to the behaviour of the *M. anjosi* adults with reduced body size and wings that facilitate their rapid locomotion within *C. fissilis* plants [14]. In addition, the evaluation period possibly favoured the rejection of the *W* value, as some individuals, randomly, may not have been captured by the sticky traps, while the low mobility of nymphs of *M. anjosi* facilitates to sample this stage by direct collection of the leaves in the field.

The number of *M. anjosi* nymphs, related to maximum and minimum temperatures and rainfall, and their relationship with the PC1 and PC2 of the PCA axes confirm an increase in their numbers in the drier months and a reduction with the temperature increases. The lower number of *M. anjosi* individuals in periods with rainfall above 100 mm is due to the relationship between accumulated rainfall and the total nymph numbers of this species as reported for *G. brimblecombei* nymphs at temperatures between 22 and 26°C and rainfall above 80 mm in Brazil [19,20,22,53]. The climate was also one of the parameters with greater impact on the population regulation of the bell bird psyllid, *Glycaspis baileyi* Moore, 1961 (Hemiptera: Psyllidae) and other psyllids species on forest red gum, *Eucalyptus tereticornis* Sm. plants in Mauritius [54].

Psyllids can have several generations per year, with all stages present, although with varying numbers between them [55]. The number of individuals of *T. limbata* increased with minimum, average and maximum temperatures and accumulated rainfall. However, the latter parameter increased the rate of vegetative growth of the plants and, consequently, the development and vigour of this psyllid due to the greater nutrient availability and leaf turgidity [56]. Torrential rains reduced arthropod populations and, by increasing soil moisture, improved the absorption of water and nutrients by inducing the emergence of new leaves on which these insects fed at the beginning of the rainy season in Costa Rica [57].

The moderate and direct correlation between natural enemies and *M. anjosi* adult numbers can be explained by the greater difficulty of these predators to capturing adults of this psyllid with reduced body size and rapid locomotion within the leaves of the *C. fissilis* plants [14].

The low relationship between the variables analysed, normality of the data and the *F* value for *M. anjosi* nymphs and adults from the analysis of main components, may be related to the restrictions on the values of the observations, strictly positive [34]. This happens with variables that appear in studies with count data, which must be integers and non-negative [58], for example, insect numbers per sample.

The sex ratio of *M. anjosi* adults in Sorocaba was similar to that of other psyllids, with amphitoky parthenogenesis, but it differs from that of the jumping plant lices, *Cacopsylla myrtilli* Wagner, 1947 [59], *Cacopsylla rare* Tuthill, 1944, *Glycaspis atkinsoni* Moore, 1984, and *Glycaspis operta* Moore, 1984 (Hemiptera: Psyllidae) [60], with thelytokous parthenogenesis and only females in their populations.

## 5. Conclusion

The number of *M. anjosi* nymphs and adults was negatively correlated with maximum and minimum temperatures and positively with relative humidity and rainfall with population peaks between the end of May and the beginning of June 2017. The high numbers of *A. opsimus*, *C. sanguinea* and *H. convergens* individuals in the yellow sticky traps and the predation of *M. anjosi* adults and nymphs on *C. fissilis* trees show the importance of these natural enemies in the integrated management of this psyllid. Yellow sticky traps were efficient in sampling of *M. anjosi* adults and its natural enemies and can be used to monitor these populations in the field. Sampling of *C. fissilis* leaves was inefficient to survey *C. sanguinea*, *H. convergens* and *A. opsimus*. The sex ratio of *M. anjosi* adults was 0.46.

## Data Availability

Primary data are provided as electronic supplementary material [61].
